# Effects of bumetanide on neurodevelopmental impairments in patients with tuberous sclerosis complex: an open-label pilot study

**DOI:** 10.1186/s13229-020-00335-4

**Published:** 2020-05-07

**Authors:** Dorinde M. van Andel, Jan J. Sprengers, Bob Oranje, Floortje E. Scheepers, Floor E. Jansen, Hilgo Bruining

**Affiliations:** 1 Department of Psychiatry, Brain Center, University Medical Center Utrecht, Utrecht, the Netherlands; 2 Department of Pediatric Neurology, Brain Center, University Medical Center Utrecht, Utrecht, the Netherlands; 3 Department of Child and Adolescent Psychiatry, Amsterdam UMC, University of Amsterdam, Amsterdam, Netherlands

**Keywords:** Tuberous sclerosis complex, Bumetanide, Open-label, NKCC1 antagonist, TAND, Irritability, ERP, Neurocognitive task

## Abstract

**Background:**

Tuberous sclerosis complex (TSC) is an autosomal dominant disease that affects multiple organs including the brain. TSC is strongly associated with broad neurodevelopmental disorders, including autism spectrum disorder symptomatology. Preclinical TSC studies have indicated altered neuronal chloride homeostasis affecting the polarity of γ-aminobutyric acid (GABA) ergic transmission as a potential treatment target. Bumetanide, a selective NKCC1 chloride importer antagonist, may attenuate depolarizing GABA action, and in that way reduce disease burden. In this open-label pilot study, we tested the effect of bumetanide on a variety of neurophysiological, cognitive, and behavioral measures in children with TSC.

**Methods:**

Participants were treated with bumetanide (2dd 0.5–1.0 mg) for 13 weeks in an open-label trial. The Aberrant Behavior Checklist-Irritability (ABC-I) subscale was chosen as the primary endpoint. Secondary endpoints included other behavioral questionnaires in addition to event-related potentials (ERP) and neuropsychological tests if tolerated. Additionally, the treatment effect on seizure frequency and quality of life was assessed. Endpoint data were collected at baseline, after 91 days of treatment and after a 28-day wash-out period.

**Results:**

Fifteen patients (8–21-years old) with TSC were included of which 13 patients completed the study. Treatment was well-tolerated with only expected adverse events due to the diuretic effects of bumetanide. Irritable behavior (ABC-I) showed significant improvement after treatment in 11 out of 13 patients (*t*(12) = 4.41, *p* = .001, *d* = .773). A favorable effect was also found for social behavior (Social Responsiveness Scale) (*t*(11) = 4.01, *p* = .002, *d* = .549) and hyperactive behavior (ABC-hyperactivity subscale) *(t*(12) = 3.65, *p* = .003, *d* = .686). Moreover, patients rated their own health-related quality of life higher after treatment. At baseline, TSC patients showed several atypical ERPs versus typically developing peers of which prepulse inhibition was significantly decreased in the TSC group. Neuropsychological measurements showed no change and bumetanide had no effect on seizure frequency.

**Limitations:**

The sample size and open-label design of this pilot study warrant caution when interpreting outcome measures.

**Conclusions:**

Bumetanide treatment is a potential treatment to alleviate the behavioral burden and quality of life associated with TSC. More elaborate trials are needed to determine the application and effect size of bumetanide for the TSC population.

Trial registration

EU Clinical Trial Register, EudraCT 2016-002408-13 (www.clinicaltrialsregister.eu/ctr-search/trial/2016-002408-13/NL). Registered 25 July 2016.

## Background

Tuberous sclerosis complex (TSC) is an autosomal dominant disorder generally caused by inactivating mutations in *TSC1* (encoding hamartin) or *TSC2* (encoding tuberin) genes [1]. The *TSC1*-*TSC2* protein complex is required for the suppression of mammalian target of rapamycin (mTOR) activity and therefore referred to as a tumor suppressor. Almost every organ can be affected in patients with TSC with hallmark features of benign tumors in vital organs including the brain [1]. As a consequence of brain involvement, TSC is strongly associated with a broad range of neurodevelopmental and psychiatric symptoms. Epilepsy is estimated to occur in 72–85% of patients [2], of which the majority responds insufficiently to antiepileptic drugs (AEDs) [3]. The broad neuropsychiatric manifestations have been denoted as *TSC-*associated neuropsychiatric disorders (TAND) [4] and comprise a range of developmental, behavioral, and affective symptoms affecting approximately 90% of TSC patients [2, 5]. The most commonly associated neurodevelopmental disorder is autism spectrum disorder (ASD), diagnosed in 40–50% of TSC patients [5]. The clinical impact of TAND is increasingly addressed by clinicians and researchers, but still, less than 20% is estimated to receive treatment for these specific symptoms [2, 5]. A large extent of TAND seems to remain unrecognized further emphasizing the need for better triage and treatment [6].

In search of treatments correcting or decreasing the neurological consequences of TSC, animal models and human remnant tissue samples from surgeries have progressed our understanding of its pathophysiology. Correction of the mTOR pathway has been a dominant theme in TSC research. Indeed, the over-activation of the mTOR signaling pathway is a direct result of the loss of *TSC1-TSC2* function in TSC. mTOR inhibitors like rapamycin analogs may, therefore, modify the TSC phenotype and several studies are currently investigating the benefit of mTOR inhibitors to treat TAND symptoms [7–9]. Yet, a recently published randomized controlled trial with mTOR inhibitor everolimus showed no effect on autistic symptoms and cognitive functioning in 4–17-year old TSC patients [10].

Another more recently proposed treatment target in TSC is chloride homeostasis. Several studies have implicated altered regulation of neuronal chloride levels in and around tubers through analysis of chloride transporter activity. More specifically, altered activity ratios between the chloride importer Na(+)-K(+)-2Cl(−) cotransporter (NKCC1) and chloride exporter Na-Cl cotransporter (KCC2) have been found [11] that may affect γ-aminobutyric acid (GABA) polarity and cause unwanted depolarizing effects of GABAergic transmission [12]. For instance, Talos and colleagues [11] showed that cortical tubers in human TSC specimens (*n* = 14), collected after surgery or post-mortem, demonstrated a decreased expression of GABA_A_α1 receptor, increased NKCC1, and decreased KCC2 levels compared to nontuberal TSC tissue and tissue from controls (*n* = 10). They additionally recorded GABA_A_R responses in cortical tissue from a single TSC patient and an epilepsy case control. The neurons from the cortical tuber slices appeared to be characterized by depolarizing GABA_A_R-mediated responses, in contrast to hyperpolarizing GABA_A_R-mediated currents in neurons from the non-TSC epilepsy case control. Ruffolo et al. [13] investigated GABAergic transmission in TSC by injecting *Xenopus* oocytes with membranes from TSC cortical tubers (*n* = 7) and control tissues (*n* = 9) at different pre- and postnatal ages. They reported that hyperpolarized GABA_A_ reversal potential was abolished in TSC tuber tissue and this was accompanied by an elevated NKCC1/KCC2 ratio in RNA expression.

These findings of altered GABAergic transmission and chloride transporter activity may constitute a treatment target to decrease disease burden in TSC [11]. Bumetanide is a selective NKCC1 antagonist and has been used as a diuretic drug for decades. In addition, bumetanide regulates neuronal chloride ion concentration by inhibiting the Na(+)-K(+)-2Cl(−) cotransporter (NKCC) and may, therefore, have therapeutic potential by reinstating hyperpolarizing GABA-activated currents [14]. In vivo cortical recordings in TSC mice (at postnatal day 16) revealed that seizure activity was blocked rapidly for over 30 min by a single parenteral administration of bumetanide [15]. Favorable clinical effects of correction of chloride homeostasis and GABAergic transmission through bumetanide has also been indicated in other experimental models of epilepsy and neurodevelopmental disorders, most notably ASD [16, 17]. In childhood epilepsy, including some specific genetic forms of epilepsy, bumetanide has been suggested to reduce seizure frequency [18, 19].

Following the available preclinical and clinical evidence for the efficacy of bumetanide in TSC and other neurodevelopmental disorders, we hypothesized that bumetanide may alleviate TAND manifestations in TSC. We conducted an open-label study to explore the effects of bumetanide on behavior, cognition, and event-related potentials (ERPs) in a sample of children and adolescents with TSC.

## Methods

The medical ethical committee (METC) of the UMC Utrecht approved the trial protocol and the study is conducted according to the principles of the Declaration of Helsinki, version of Fortaleza, 2013, the International Conference on Harmonisation–Good Clinical Practice (ICH-GCP) and in accordance with the Medical Research Involving Human Subjects Act (WMO). Written informed consent was obtained from all parents and participants.

### Participants

Male and female TSC patients were recruited via the database of the TSC center of excellence of the Department of Pediatric Neurology of the UMC Utrecht and via online advertisement on the website of the Dutch TSC patient organization (https://stsn.nl/). Potential participants were screened for eligibility by a child psychiatrist (HB) and child neurologist (FJ). Children with a definite TSC diagnosis based on genetic or clinical diagnostic criteria (as established by the 2012 International Tuberous Sclerosis Complex Consensus Conference [20]), between 8 and 21 years old, and > 30 kg were eligible as participants. Patients with intellectual disability (ID [TIQ ≤ 70]) and without ID (IQ > 70) were both included, to study a representative sample. The use of concomitant antiepileptic and psychoactive drugs was allowed, when being taken on a stable regime at least 8 weeks prior to baseline. Exclusion criteria were renal and liver insufficiencies, serious unstable illnesses (including gastroenterological, respiratory, cardiovascular, endocrinologic, immunologic, hematologic disease, dehydration or hypotension, electrolyte disturbances), treatment with NSAIDS, aminoglycosides, digitals, antihypertensive agents, indomethacin, probenecid, acetazolamide, lithium, other diuretics, stimulants (like methylphenidate and dexamphetamine), and drugs known to have a nephrotoxic potential.

To compare ERP measures of our sample at baseline with typically developing (TD) children, data from a TD control group collected at the department of psychiatry at the UMC Utrecht between 2015 and 2018 consisting of 39 children (49% male) aged 7–15 years old (M = 12.9; SD = 3.8) with no history of medical or developmental or learning problems (TIQ M = 118.2; SD = 14.6) was used. This control group was tested in the same EEG lab using identical ERP paradigms and conditions.

### Design

All participants and their parent(s) visited the outpatient Psychiatry department of the UMC Utrecht for a screening visit and baseline measurements between March 2017 and April 2018. This visit included a detailed interview of medical and family history (to complete the data already collected in the database and medical records), a physical examination, blood and urine analysis, and if obtainable, an IQ-estimation assessment. The total study assessment period consisted of 8 to 11 study visits: visit 1 (week 4, screening and baseline assessment), visit 2–3 (neuropsychological testing and EEG on separate days), visit 4 (day 4), visit 5 (day 7), visit 6 (day 14), visit 7 (day 28), visit 8 (day 56), visit 9–10 (day 91, end of treatment and neuropsychological measurements and EEG), and visit 11 (day 119, end of wash-out and EEG).

### Treatment

Patients were treated with bumetanide CF 1.0 mg tablets (RVG 23140) for 91 days, as add-on treatment. They received 0.5 mg (i.e., half a tablet) bumetanide twice daily (breakfast and afternoon) as starting dosage, which was increased to 1.0 mg twice daily if blood electrolytes were normal at visit day 7. This dose was selected as this presented the most favorable benefit/risk ratio in the phase IIB bumetanide RCT (*n* = 88) for children with neurodevelopmental disorders (i.e., ASD) [21]. Due to expected hypokalemia, all participants received oral potassium-chloride supplements. To evaluate the tolerability and safety of bumetanide in TSC, blood analysis, physical examination of vital signs, epilepsy diary assessment, and report of adverse events were carried out on day 4, 7, 14, 28, and 56. Blood analysis included sodium, potassium, chloride, uric acid, urea, creatinine, glucose, estimated glomerular filtration rate, hematocrit, hemoglobin, erythrocytes, leukocytes, thrombocytes, and total protein. On day 91 and 119 only physical examination was carried out.

### Endpoint measurements

#### Behavioral and quality of life (QOL) questionnaires

Clinical endpoint questionnaires included the Aberrant Behavior Checklist (ABC) [22], Social Responsiveness Scale-2 (SRS) [23], Sensory Profile-2 (SP-NL) [24], Sensory Profile School Companion (SP-SC) [25], Repetitive Behavior Scale-Revised (RBS-R) [26], and Behavior Rating Inventory of Executive Function (BRIEF; versions parent and teacher-reported) [27]. In addition to these validated questionnaires, the TAND checklist [5] was administered during an interview for additional evaluation of the broad TSC-related psychiatric manifestations. Although the checklist was originally developed as a screening tool to describe and evaluate the multidimensional TAND symptoms, we quantified the prevalence of TAND symptoms on the basis of parental-reported incidence. Parent’s QOL was assessed by the World Health Organization QOL (WHOQOL–BREF) [28] and EQ-5D-5 L [29] whereas the EQ-5D-Youth [30] and Pediatric Quality of Life Inventory (PedsQL) [31] focused on health-related QOL of the patients. In order to provide a meaningful interpretation of the data, we compared raw scores instead of normative data (i.e., not corrected for calendar age).

#### ERP measurements

Neurophysiological effects of treatment were measured using electroencephalography (EEG) to assess automatic responses to auditory stimuli (paradigms have previously been described as part of the Copenhagen Psychophysiological Test Battery [32–34]. Prepulse inhibition of the startle reflex paradigm (PPI) to measure sensorimotor gating, a P50 suppression task to measure sensory gating, and a mismatch negativity (MMN) oddball paradigm was used to evaluate automatic auditory discrimination. All measurements took place in the morning and participants were asked to refrain from consuming caffeinated beverages and foods, albeit being allowed to take their regular medication. Participants were seated and requested to sit still while auditory stimuli were presented through tubal insert earphones (EARtone®, Etymotic Research) by a computer running Presentation® software (Neurobehavioral System Inc.). For details about the ERP paradigms, acquisition and (pre) processing see Additional file 1. Not all participants were capable of understanding the instructions and cooperating in the ERP assessment; a total of 9/13 patients underwent ERP measurements.

#### Neuropsychological measurements

Attentional, memory, and flexibility skills were tested, as these areas were considered of particular concern according to the consensus clinical guidelines for the assessment of cognitive and behavioral problems in TSC [35]. The battery included a baseline reaction time task, a Go/No-Go task, different memory tasks, and an auditory and visual set shifting task (Amsterdam Neuropsychological Test Battery [36]). A total of 7/13 patients completed all neuropsychological measurements.

### Statistical analyses

Statistical analyses were performed using IBM SPSS Version 25.0. Descriptive analyses, Kolmogorov-Smirnov tests, and histograms were used to identify the distribution of outcome responses on behavioral questionnaire data. When data were normally distributed, paired-samples Student’s *t* tests were used to compare results before and after treatment (i.e., D0 versus D91); otherwise, Wilcoxon signed-rank tests were used. Comparisons between D0 and D119 and D91 and D119 were made to explore possible alterations after wash-out. Wilcoxon singed-rank tests were used to compare the patients’ EEG-data between these time points and Mann-Whitney tests (exact statistic) to compare TSC patients with the TD control group at baseline. Tests were two-sized and *p* values < .05 were considered significant for main analyses. Bonferroni corrections (1 − (1 − α)^1/n^) were applied when multiple comparisons across secondary behavioral subscales or ERP parameters were performed. Effect sizes for significant effects were calculated using Cohen’s *d* [37] with the following formulas respectively for within-subject (treatment effect) and between-subject effects (group difference ERP measures): $d=\frac{\left|m1-m2\right|}{\sqrt{s_1^2+{s}_2^2-\left(2{rs}_1{s}_2\right)}}$ and $d=\frac{\left|m1-m2\right|}{\sqrt{\frac{\left({n}_1-1\right){s}_1^2+\left({n}_2-1\right){s}_2^2}{n_1+{n}_2-2}}}$ Due to the limited sample size, exploratory analyses were used for neuropsychological data.

## Results

### Cohort characteristics

**Fig. 1:**
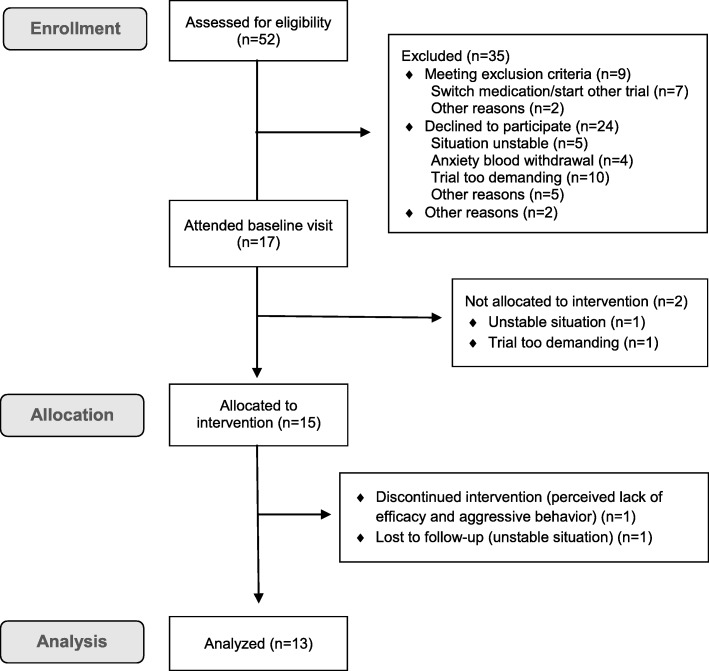
Flow diagram of BATSCH-study

Fifty-two potential participants were screened for eligibility by telephone and 17 patients who met inclusion criteria consented and attended the baseline visit (Fig. 1). After this visit, 15 patients enrolled in the study and started treatment. One patient was lost to follow-up (after completing 91 days treatment) and one patient dropped out at day 50 due to aggressive behavior. Thus, a total of 13 patients completed the study, see Table 1 for characteristics. Nine were able to comply with EEG assessment and in 7 cognitive functioning could reliably be assessed.

**Table 1:** Cohort characteristics

Patient	Age	Sex	TIQ	Gene	Mutation	Epilepsy	Seizure control^1^	Medication	Psychiatric diagnoses^2^
1	8.3	M	82	TSC-2	De novo	Focal (M)	Yes	VPA, OXC	–
2	9.4	F	<40	TSC-2	De novo	Focal (N-M)	No	VPA, LTG, VGB	ASD
3	9.5	M	107	TSC-1	Familial	No	NA	NA	ASD
4	10.1	M	<40	TSC-2	De novo	Focal (M)	No	VPA, FBM	ASD
5	10.7	M	107	NMI	NA	Focal (M)	Yes	OXC	–
6	11.2	M	47	TSC-2	Familial	Focal (M)	Yes	VPA	ASD
7	11.8	F	80	TSC-1	Familial	No	NA	NA	ASD
-	12.6	M	49	NMI	NA	Focal (M)	Yes	LEV, RAM	–
8	13.7	F	88	TSC-1	Familial	CR	NA	NA	–
9	13.8	F	51	NT	NA	Focal (M)	No	OXC	ADD, MD
-	14.0	F	63	TSC-2	De novo	Focal (M)	Yes	OXC	Selective mutism
10	14.4	M	<40	TSC-2	De novo	Focal (N-M)	No	VPA	ASD
11	16.3	F	70	TSC-2	De novo	No	NA	ESC	ASD, MD
12	17.6	F	88	TSC-2	Familial	No	NA	ESC	Anxiety, MD
13	21.3	M	49	TSC-2	De novo	Focal (M)	No	LEV	–

*Abbreviations*: *ASD* autism spectrum disorder, *F* female, *M* male, *TIQ* total intelligence quotient, *NA* not applicable, *NT* not tested, *NMI* no mutation identified, *CR* complete remission, *ESC* escitalopram, *OXC* oxcarbazepine, *VPA* valproic acid, *FBM* felbamate, *LEV* levetiracetam, *LTG* lamotrigine, *VGB* vigabatrin, *RAM* Ramipril, *N-M* non-motor, *MD* mood disorder

Note: The dashes in the first column represent 2 patients that did not complete the study and were not included in analyses

^1^Seizure-free for > 1 year

^2^Expert clinical diagnoses

### Behavioral questionnaires

Following the 3-month bumetanide treatment, we found a significant reduction of clinical and behavioral symptoms as measured with several endpoint questionnaires. A significant effect was obtained for the primary endpoint, the Aberrant Behavior Checklist Irritability subscale (ABC-I) score, indicating a reduction of irritable behavior after treatment (D0: M = 14.2, SD = 7.6 versus D91: M = 8.3, SD = 7.5; *t*(12) = 4.41, *p* = .001, *d* = .773). This effect persisted after wash-out (D0: M = 14.2, SD = 7.6 versus D119: M = 8.8, SD = 6.2; *t*(12) = 3.81, *p* = .002, *d* = .776) with no change observed between D91 and D119 (D91 versus D119; *t*(12) = -3.93, *p* = .701). Improvement in hyperactive behavior was observed through the reduction of the hyperactivity subscale of the ABC (ABC-H) (D0: M = 11.6 SD = 7.1 versus D91: M = 7.2, SD = 5.6; *t*(12) = 3.65, *p* = .003 [adjusted significance level *p* < .0125], *d* = .686). No improvement was noted on the other ABC-subscales (i.e., lethargy, stereotyped behavior, and inappropriate speech: *p* > .137; *d* < .333).

**Fig. 2:**
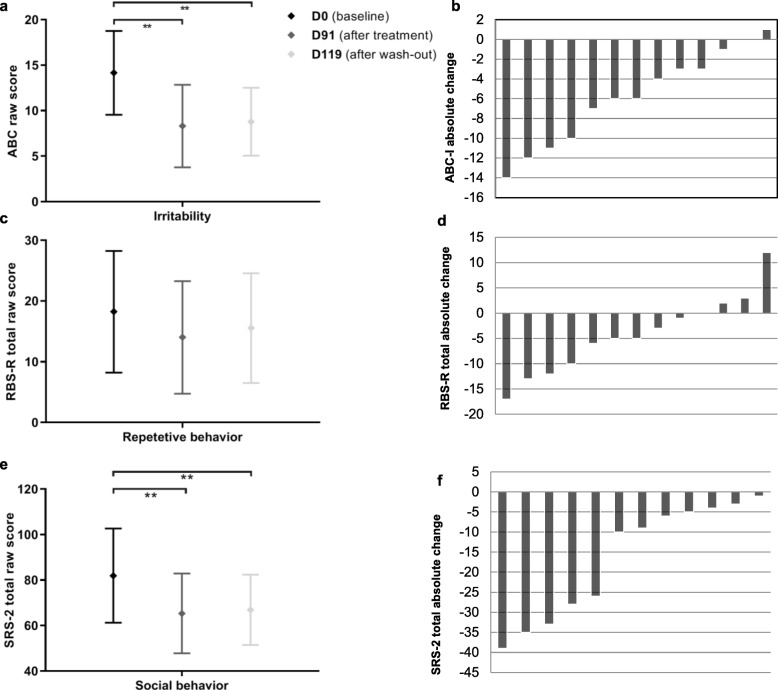
Treatment effect measured by behavioral questionnaires. Left panes show change after treatment and wash-out for (**a**) ABC Irritability subscale; (**c**) RBS total score; and (**e**) SRS-2 total score. Right panes show absolute change per patient after treatment for (**b**) ABC Irritability subscale; (**d**) RBS total score; and (**f**) SRS-2 total score

The RBS-R total score indicated no change in repetitive behavior (*Z* = −1.885; *p* = .059). Although an improvement in compulsive behavior was indicated on the RBS-R subscale “compulsive behavior”, this did not survive multiple correction (*Z* = −2.448; *p* = .014 [adjusted significance level *p* < .0083]). Social behavioral improvements through treatment were observed on total scores of the SRS-2 (D0: M = 81.9, SD = 32.6 versus D91: M = 65.3, SD = 27.6; *t*(11) = 4.01, *p* = .002, *d* = .549). This improvement persisted after wash-out (D0: M = 81.9, SD = 32.6 versus D119: M = 66.9, SD = 24.3; *t*(11) = 3.27, *p* = .007, *d* = .522); and showed no difference between D91 and D119 (*t*(11) = −.54, *p* = .598). Furthermore, an improvement was observed on the SRS-2 subscale “social communication” (D0: M = 24.8, SD = 12.9 versus D91: M = 18.7, SD = 12.0; *t*(11) = 4.65, *p* < .001 [adjusted significance level *p* < .01], *d* = .481), but did not survive multiple corrections on the following subscales: “social cognition” (D0: M = 17.7, SD = 6.3 versus D91: M = 15.2, SD = 5.3; *t*(11) = 2.28, *p* = .044 [adjusted significance level *p* < .01]), “social awareness” (*Z* = 2.044; *p* = .041 [adjusted significance level *p* < .01]) and “autistic preoccupations” (*Z* = 2.587; *p* = .01 [adjusted significance level *p* < .01]). Figure 2 provides an overview of the treatment effect measured by the ABC, SRS-2, and RBS-R questionnaires.

To assess effects on sensory processing difficulties, quadrant and section (A-D) scores of the Sensory Profile-2 (SP-NL) were analyzed, showing no change after treatment (*p > .013* [adjusted significance level *p* < .0125]). Finally, the parent-reported Behavior Rating Inventory of Executive Function (BRIEF) showed improvement after treatment (D0: M = 142.8, SD = 20.2 versus D91: M = 130.1, SD = 18.9; *t*(9) = 3.125, *p* = .012, *d* = .649), although the questionnaire could be analyzed in only 10 patients as many items were rated as “not applicable”. The SP School Companion and teacher-reported BRIEF could not be analyzed due to many missing observations.

**Fig. 3:**
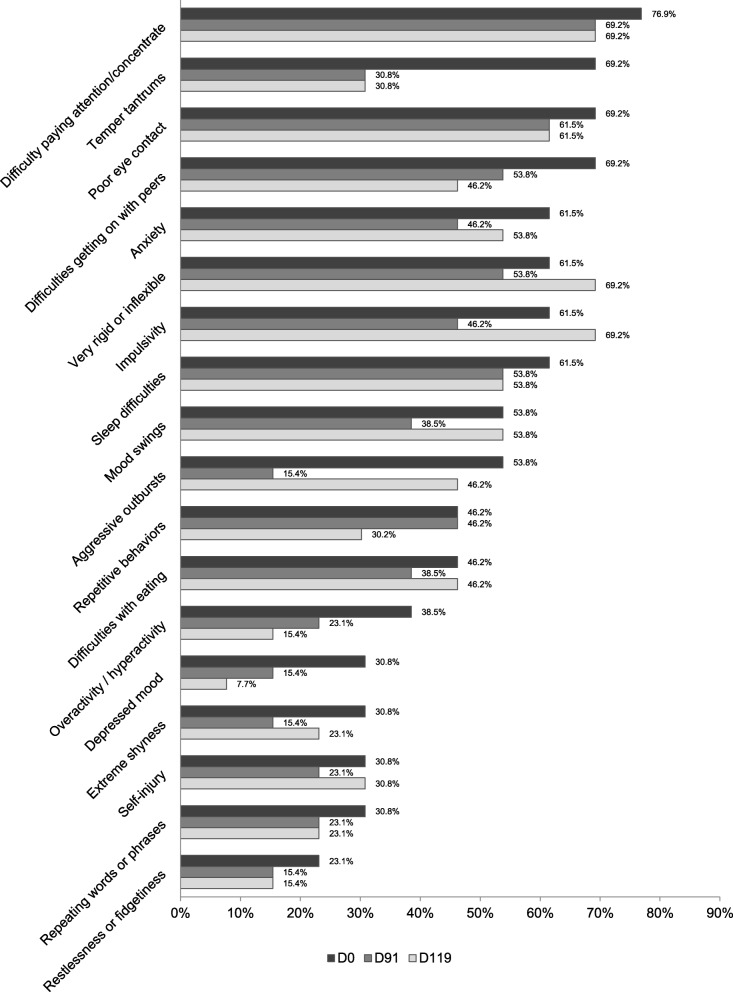
Percentage of parents reporting concerns/difficulties from section 3 of the TAND checklist at baseline (D0), after treatment (D91), and after wash-out (D119)

In addition to the validated questionnaires, we also applied the TAND checklist before and after treatment to expand the exploration of possible effects on other psychiatric manifestations associated with TSC. Figure 3 shows the percentages of reported symptoms at baseline, after treatment and wash-out. At baseline, the most reported symptoms in the behavioral dimension of TAND were difficulty paying attention or concentrating (76.9%), temper tantrums (69.2%), poor eye contact (69.2%), difficulties getting on with other people of similar age (69.2%), anxiety, very rigid or inflexible about how to do things or not liking the change in routines (69.2%), impulsivity (69.2%), and sleep difficulties (69.2%).

**Fig. 4:**
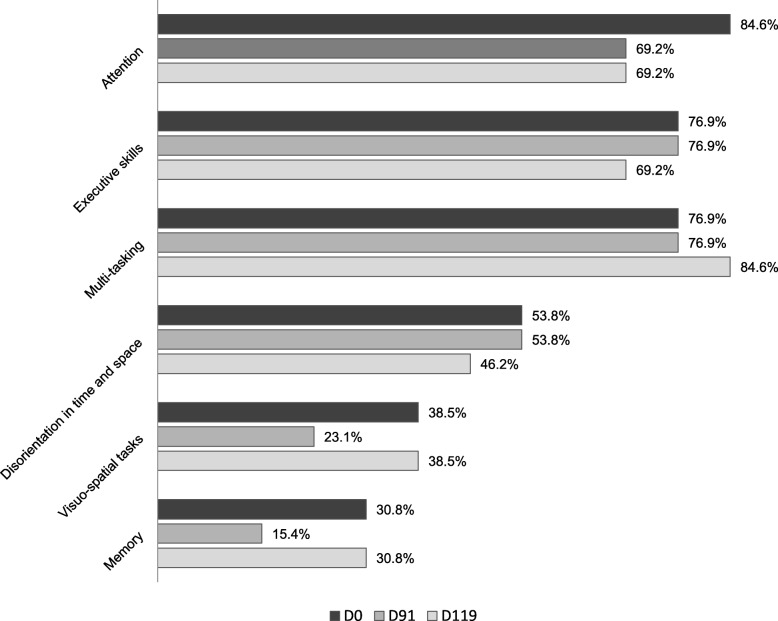
Percentage of parents reporting difficulties in cognitive skills from section 7 of the TAND checklist at baseline (D0), after treatment (D91), and after wash-out (D119)

After treatment, substantially less temper tantrums (−38.4) and aggressive outbursts (−38.4) were reported. Repetitive behavior did not improve. On the cognitive dimension (section 7), parents reported most problems to be apparent in attention (84.6%), executive skills (76.9%), and multi-tasking (76.9%) (see Fig. 4) but no improvements were found after treatment.

#### Quality of life questionnaires

Patient-reported scales showed that a significantly higher quality of life score on the EQ-5D-Youth was given after treatment (D0: M = 69.5, SD = 12.3 versus D91: M = 77.0, SD = 11.1; *t*(9) = −2.42, *p* = .038). The self and proxy-reported subscales of the PedsQL (i.e., physical functioning, emotional functioning, social functioning, and school functioning) showed no change after treatment. Before treatment, parents rated their own general health on the 0–100 scale of the EQ-5D-5 L with an average of 81.3 (fathers) and 81.1 (mothers), which remained stable after their child participated in the trial (81.1 and 80.4, respectively; *n* = 12). Reports in the 4 domains of the WHOQOL-BREF (i.e., physical health, psychological, social relationships, and environment) also remained stable during treatment.

### ERP measurements

#### PPI

**Fig. 5:**
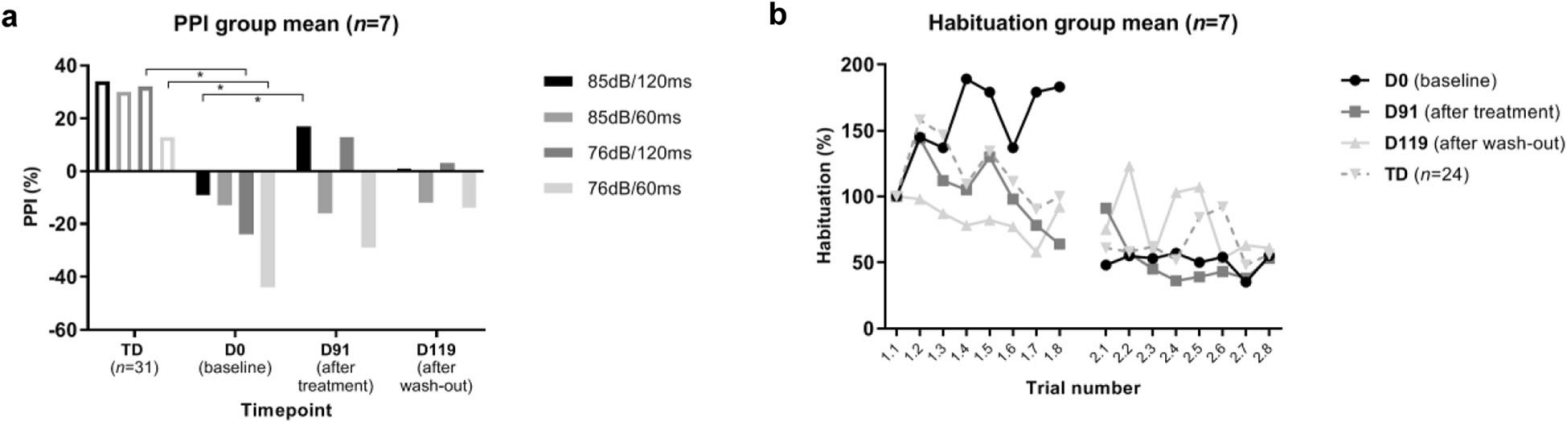
**a** Mean percentage PPI in the TD group and TSC patients for all four prepulse-pulse trials. Significantly less PPI was found in TSC compared with TD in the two 76 dB types at baseline. Improvement was found after treatment in trial-type 85 dB/120 ms. **b** Startle amplitude measured with electromyography for the 8 trials of blocks 1 and 2 in a habituation paradigm. Increased sensitization is apparent in the TSC group at baseline and habituation changes after treatment (D91) and wash-out (D119)

Table 2 and Fig. 5a show the main results from the PPI paradigm—assessing sensorimotor gating—with four prepulse-pulse trials. At baseline, the TSC group showed only prepulse facilitation instead of inhibition: all trial types, therefore, showed decreased PPI in TSC patients compared with TD controls (76 dB/120 ms *U* = 35, *p* = .004, *d* = −1.643; 76 dB/60 ms: *U* = 39, *p* = .007, *d* = −1.188; 85 dB/120 ms: *U* = 46, *p* = .017, and 85 dB/60 ms: *U* = 51, *p* = .03), although this only survived Bonferroni correction in the two 76 dB trial types, not in the two 85 dB trial types (adjusted significance level *p* < .0125). The habituation coefficient showed no difference between groups at baseline (*U* = 47, *p* = .650; see Table 2). However, where the TD group showed habituation starting from the third trial in block 1, the TSC group only showed sensitization (meaning an increase in startle amplitude compared with the first pulse alone trial) within the first habituation block, albeit not surviving multiple corrections when comparing individual trials (trial 4: *U* = 37, *p* = .026, and trial 7: *U* = 36, *p* = .022; adjusted significance level *p* < .0125).

**Table 2:** Habituation and percentage prepulse-pulse inhibition (PPI)

	TD (***n*** = 31)	TSC (***n*** = 7)		
Time point	D0	D0	D91	D119
Trial	Mean % (SD)	Mean % (SD)		
85dB/120ms	34 (33)	-9 (52)	**19 (25)**^c^	1 (55)
85dB/60ms	30 (35)	-13 (45)	-15 (16)	-12 (45)
76dB/120ms	**32 (27)**^ab^	-24 (58)	13 (30)	3 (21)
76dB/60ms	**13 (49)**^ab^	-44 (55)	-26 (34)	-14 (20)
Habituation coefficient	-4.52	-1.91	**-6.63**^ac^	**-.78**^ac^

*Abbreviations*: *TD* typically developing controls, *TSC* tuberous sclerosis complex

^a^significant after Bonferroni correction; ^b^group effect; ^c^treatment effect

Treatment with bumetanide showed a tendency to reduce prepulse facilitation for both 120 ms trials, which only reached significance for the 85 dB/120 ms trial type, but did not survive Bonferroni correction (*Z* = 2.45, *p* = .014, adjusted significance level *p* < .0125). No change was observed in the other PPI trials (*p* > .140). Figure 5b shows the average startle amplitudes in the habituation paradigm. Visual inspection suggested normalization of habituation after treatment although this did not reach statistical significance (*Z* = 1.214, *p* = .225). There was a significant difference between habituation after treatment and wash-out (*Z* = 2.366, *p* = .016, *d* = .773).

#### P50

**Fig. 6:**
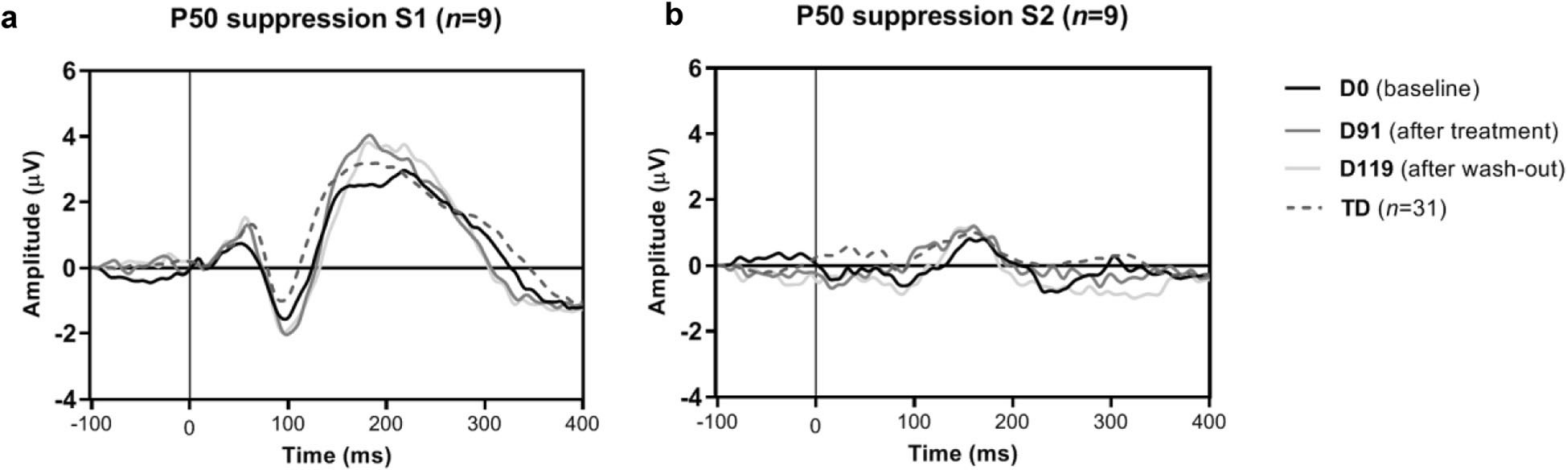
Grand average of lead Cz of the P50 suppression paradigm, for the a) conditioning (“S1”) and b) testing stimuli (“S2”) showing no difference between group and time points

Table 3 and Fig. 6 show the grand averages for the P50 suppression paradigm—assessing sensory gating. No baseline differences were found between the TSC and TD group for the S1 amplitude (*U* = 122.5, *p* = .593), S2 amplitude (*U* = 137.5, *p* = .956), and the S1/S2 ratio (*U* = 110.5, *p* = .357). After treatment or wash-out, no changes were found on the S1 amplitude, S2 amplitude, and S1/S2 ratio (*p* > .123).

**Table 3:** P50 suppression amplitudes of S1, S2, and S1/S2 and latencies at different time points

	TD (***n*** = 31)	TSC (***n*** = 7)		
Time point	D0	D0	D91	D119
Trial	Mean amplitude (SD)	Mean amplitude (SD)		
S1 amplitude	1.65 (1.01)	1.42 (.68)	1.98 (1.26)	1.67 (1.38)
S1 latency	61.35 (10.39)	62.67 (11.40)	63.33 (9.06)	62.67 (9.95)
S2 amplitude	1.00 (1.04)	.88 (.70)	1.02 (.89)	.74 (.52)
S2 latency	59.27 (15.45)	65 (9.97)	67.75 (14.44)	66.22 (14.12)
S1/S2 ratio	.58 (.55)	.73 (.50)	.81 (.85)	.65 (.52)

*Abbreviations*: *TD* typically developing controls, *TSC* tuberous sclerosis complex

#### MMN

**Fig. 7:**
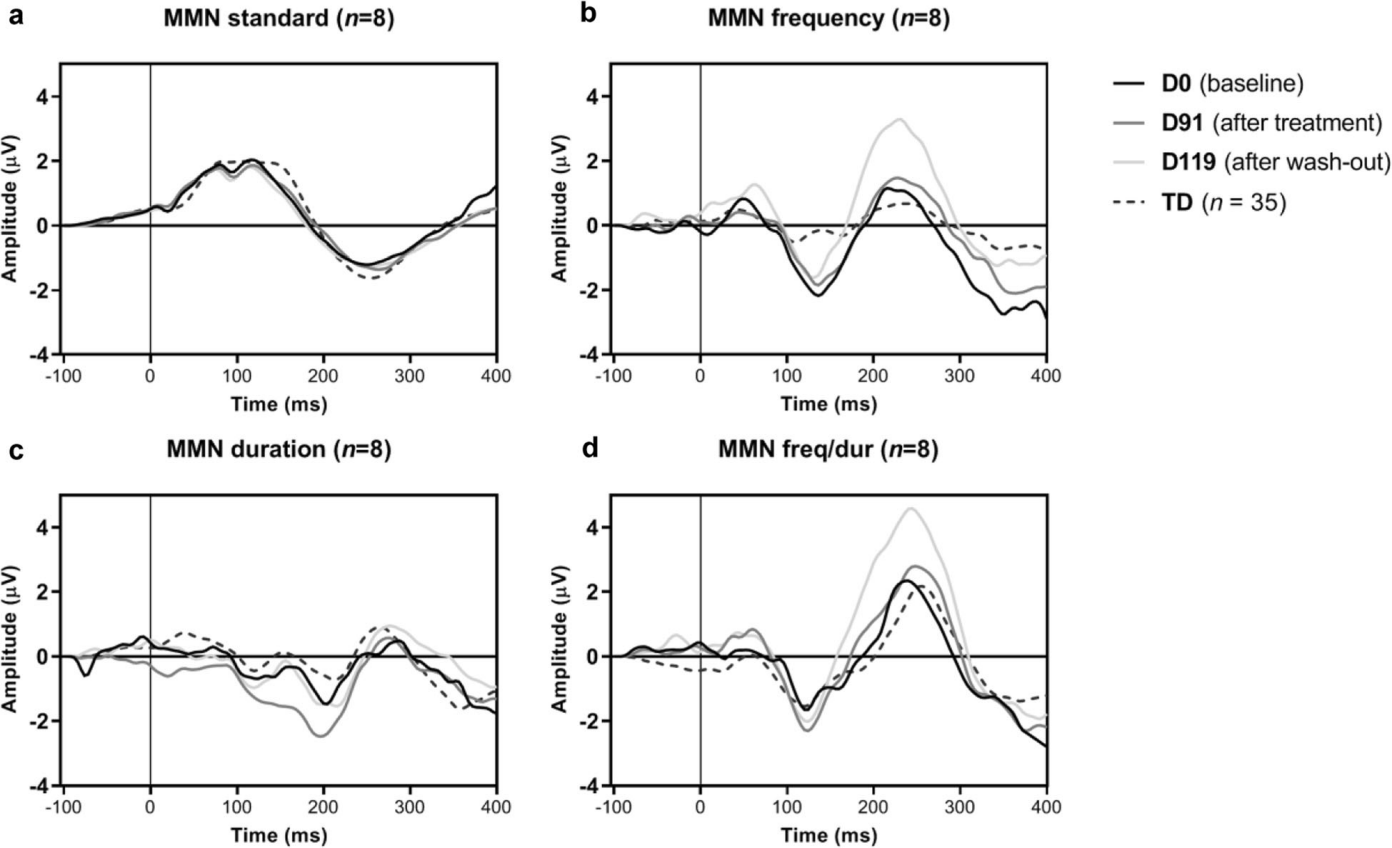
Grand averages of lead FCz of the mismatch negativity paradigm, for the (**a**) standard stimulus; (**b**) frequency deviant; (**c**) duration deviant, and (**d**) frequency/duration deviant. Dashed lines represent data from the typically developing control group (*n* = 35)

Table 4 and Fig. 7 present the grand averages of the mismatch negativity paradigm, evaluating automatic auditory discrimination, for the four different stimuli (i.e., standard, frequency deviant, duration deviant, and frequency/duration deviant). Compared with TD, the TSC group at baseline showed enhanced automatic discrimination of frequency deviant tones (*U* = 53, *p* = .005 [adjusted significance level *p* < .0125], *d* = .893). For the other deviant tones, no significant difference was found at baseline (*U* > 127, *p >* .702). The only effect after treatment on MMN was found for duration MMN; which was enhanced compared to baseline (D0 vs D91: *Z* = 1.96, *p* = .05, *d* = 1.061) and returned to baseline values after wash-out (D0 vs D119: *Z* = .56, *p* = .575). The MMN due to the other deviants showed no difference between treatment time points (*p* > .161) meaning the increase in frequency MMN persisted after treatment and wash-out.

**Table 4:** Mismatch negativity (MMN) mean amplitudes and latencies

	TD (***n*** = 35)		TSC (***n*** = 8)					
Time point	D0		D0		D91		D119	
Deviant type	Amp (SD)	Lat (SD)	Amp (SD)	Lat (SD)	Amp (SD)	Lat (SD)	Amp (SD)	Lat (SD)
Standard	−1.82 (.92)	255 (21)	−1.45 (.46)	253 (34)	−1.51 (.52)	261 (12)	−1.65 (.74)	258 (34)
Frequency	−**1.68 (1.22)**^ab^	138 (40)	−**3.03 (1.75)**^a^	137 (23)	−2.75 (1.71)	136 (28)	−2.23 (.70)	125 (28)
Duration	−1.91 (1.31)	193 (49)	−**1.71 (1.5)**^c^	206 (28)	−**3.11 (1.10)**^c^	206 (31)	−1.92 (1.15)	203 (20)
Freq/Dur	−2.35 (1.15)	130 (29)	−2.28 (1.32)	125 (34)	−2.64 (1.52)	116 (16)	−2.46 (1.19)	122 (25)

*Abbreviation*: *MMN* mismatch negativity, *Amp* amplitude, *Lat* latency, *SD* standard deviation, *Freq/Dur* frequency duration deviant, *TD* typically developing control group

^a^Significant with Bonferroni correction; ^b^group effect;^c^treatment effect

### Neuropsychological measurements

No changes were observed with neuropsychological measurements after treatment. Additional file 2 shows all results obtained with the different neuropsychological tests.

### Safety and tolerability

No serious adverse events (SAEs) were reported during the trial. Bumetanide was well tolerated by the majority of patients. As expected from the diuretic effect, hypokalemia was the most commonly observed adverse event, in spite of oral potassium-chloride supplements. Table 5 provides an overview of all reported adverse events in all patients enrolled and the (expected) relationship with the investigational product. Details on the blood safety analyses (i.e., potassium and sodium) are provided in Additional file 3.

**Table 5:** Adverse events

Event	Severity	Intervention relationship^a^	Expected	# Participants
**Blood analyses**				
Leukocytopenia	Mild	2-3	Yes	1
Hypokalemia	Mild	1-2	Yes	7
Hyponatremia	Mild	2	Yes	1
Hypovolemia	Mild	1	Yes	1
**Gastrointestinal symptoms**				
Nausea/vomiting	Mild	3	Yes	1
Obstipation	Mild	2	Yes	2
Decreased appetite	Mild	2	No	1
Weight loss	Mild	2	Yes	1
**Genitourinary**				
Diuresis	Mild	1	Yes	1
**Infections**				
Viral infection	Mild	3	No	1
Urinary tract infection	Mild	2	No	1
**Behavioral symptoms**				
Aggression	Moderate	2	No	2
Irritability	Mild	2	No	2
Anxiety	Mild	2	No	1
**Musculoskeletal**				1
Medial malleolus fracture	Moderate	3	No	1
Humerus fracture	Moderate	3	No	1
**Other**				
Hypotension	Mild	1	Yes	1
Hypothermia	Mild	3	No	1
Dehydration	Mild	1	Yes	1
Palpitations	Mild	2	No	1

Note: ^a^1: Definitely related; 2: Possibly related; 3: Not related

### Seizure frequency

Seizure frequency did not change after 3 months of bumetanide treatment. Changes in seizure frequency from D-28 to D91 are depicted in Additional file 4 for all patients with uncontrolled epilepsy.

## Discussion

This open-label pilot study tested the effect of 91 days of bidaily 0.5–1.0 mg bumetanide treatment on behavior, cognition, and ERP parameters in a sample of children and adolescents with TSC. In general, bumetanide was well tolerated by the majority of participants and no serious adverse events occurred. In this sample, we found significant improvement in the primary endpoint, the aberrant behavior checklist (ABC)—irritability subscale. Another main finding included a significant improvement in social behavior (SRS-2). Beneficial effects of bumetanide were also indicated through quality of life assessments: patients rated their own health-related quality of life significantly higher after bumetanide treatment, together with parents reporting fewer problems with self-esteem in their children.

Although these are promising results, we should acknowledge that this is an open-label study with a limited sample size. However, many parents noticed strong amelioration of behavioral manifestations as expressed in the preliminary evaluations made with the TAND checklist and substantiated by the ABC-I, most notably improvements in the number and duration of temper tantrums and aggressive outbursts. Another main parental concern according to the TAND checklist were social symptoms, such as difficulties getting on with peers and poor eye contact—which indeed improved according to SRS-2 results. In addition, according to the TAND-checklist and QoL questionnaires the familial situation also seemed to improve, showing a reduction of familial stress and improved relationships between parents through treatment. These results may be consistent with previously reported effects of bumetanide on autism spectrum disorder (ASD) symptomatology [21, 38, 39], a neurodevelopmental disorder strongly associated with TSC.

Using the ERP analyses, we found changes between TD and TSC samples for PPI and MMN both at baseline and through treatment. This may support the hypothesis that the effect of bumetanide on TAND may be mediated through alterations in neurophysiology.

The origins of the ERPs changes in TSC are complex to interpret since MMN may also be sensitive to (localized) epileptic activity and antiepileptic treatment [40, 41]. However, we do expect these effects to be limited because the dosage of antiepileptic treatment was stable prior and during the study. To our knowledge, no other trials have studied the effect of bumetanide on ERPs. It should be noted that ERP parameters are variable even in a genetic disorder as TSC perhaps due to differences in phenotypic expression including brain malformations. Both the neuropsychological test battery as well as the parent-reported cognitive skills (TAND checklist) indicated no changes in cognitive functioning through treatment.

## Limitations

As mentioned, an important limitation of this study is the single-arm open-label design and the absence of a placebo group. TSC is a rare genetic disorder and patients often experience extensive physical burden and unstable disease courses; therefore, recruitment in placebo-controlled trials is highly challenging, with subsequently small sample sizes, insufficient to draw firm conclusions. Although a TD control group with a similar mean age (i.e., 12.9 years) was included to compare ERP measurements, this group had a more narrow age range (7–15 years) and no repeated EEG measurements were performed. We could only administer our testing battery in patients with an IQ above 70; therefore, we cannot extrapolate the cognitive and ERP findings to TSC patients with intellectual disability. Another caveat may be that 91 days of bumetanide is not long enough to mediate significant effects on cognitive function tests or effects are too subtle to detect with our test battery in the exceptional context of a hospital environment. Researchers testing everolimus in TSC have also suggested that age of administration is an important factor in the treatment of TAND symptoms [9], as TAND symptoms initially present early (i.e., within 2 years of age) in life. Another limitation is that assessment of TSC and epilepsy-specific symptoms could have been carried out by using more sensitive outcome measures, such as using the quality of life in childhood epilepsy (QOLCE) and Quality of Life in Epilepsy Inventory for Adolescents-48 (QOLIE-AD-48), which has shown good psychometric properties [42]. An additional uncontrolled factor is the variety of co-medication used in 11 out of 14 patients, complicating the interpretation of the direct effects of bumetanide. It should be noted that the dosage of co-medication was kept stable during the study period. It is not possible yet to measure neuronal chloride concentrations in the clinical situation, thus it remains unclear whether treatment effects are exerted via correction of chloride homeostasis. Moreover, studies have shown that bumetanide may have limited penetrance in the brain so that we cannot rule out that peripheral effects also contribute to the observed changes [43].

Despite these limitations, this study indicates a favorable effect of bumetanide in TSC on clinically important behavioral symptoms. These findings may be followed up in more elaborate studies either in a larger multicenter randomized controlled design or a multiple *n* = 1 design (e.g., [44] given the rarity of the genetic disorder.

## Conclusion

This pilot study indicates the potential efficacy of bumetanide on behavioral problems in young patients with TSC. Bumetanide improved irritable, explosive, and social behavior in the majority of patients in this sample and treatment was well tolerated.

## Supplementary information

**Additional file 1.** Supplementary methods.

**Additional file 2.** Neuropsychological test battery results.

**Additional file 3.** Blood safety checks.

**Additional file 4.** Seizure frequency.

## Data Availability

The data that support the findings of this study are available from the Psychiatry department of the UMC Utrecht and can be made available from the authors upon reasonable request and with permission of HB and FS.

## Abbreviations

ABC: Aberrant behavior checklist
ASD: Autism spectrum disorder
BRIEF: Behavior rating inventory of executive function
EEG: Electroencephalography
ERP: Event-related potentials
ESC: Escitalopram
FBM: Felbamate
GABA: γ-aminobutyric acid
ID: Intellectual disability
LTG: Lamotrigine
LEV: Levetiracetam
MMN: Mismatch negativity
mTOR: Mammalian target of rapamycin
NKCC: Na(+)-K(+)-2Cl(−) cotransporter
OXC: Oxcarbazepine
PPI: Prepulse inhibition
QOL: Quality of life
RAM: Ramipril
RBS: Repetitive behavior scale
SP: Sensory profile
SRS: Social responsiveness scale
TAND: TSC-associated neuropsychiatric disorders
TD: Typically developing
TSC: Tuberous sclerosis complex
VBG: Vigabatrin
VPA: Valproic acid

## References

[1] Curatolo P, Bombardieri R, Jozwiak S (2008). Tuberous sclerosis. Lancet.

[2] Curatolo P, Moavero R, de Vries PJ (2015). Neurological and neuropsychiatric aspects of tuberous sclerosis complex. Lancet Neurol.

[3] Chu-Shore CJ, Major P, Camposano S, Muzykewicz D, Thiele EA (2010). The natural history of epilepsy in tuberous sclerosis complex. Epilepsia.

[4] Krueger DA, Northrup H, Group ITSCC (2013). Tuberous sclerosis complex surveillance and management: recommendations of the 2012 International Tuberous Sclerosis Complex Consensus Conference. Pediatr Neurol.

[5] de Vries PJ, Whittemore VH, Leclezio L, Byars AW, Dunn D, Ess KC (2015). Tuberous sclerosis associated neuropsychiatric disorders (TAND) and the TAND Checklist. Pediatr Neurol.

[6] Kingswood JC, d’Augères GB, Belousova E, Ferreira JC, Carter T, Castellana R (2017). TuberOus SClerosis registry to increase disease Awareness (TOSCA)—baseline data on 2093 patients. Orphanet Journal of Rare Diseases.

[7] de Vries PJ (2010). Targeted treatments for cognitive and neurodevelopmental disorders in tuberous sclerosis complex. Neurotherapeutics.

[8] Randell E, McNamara R, Davies DM, Owen-Jones E, Kirby N, Angel L (2016). The use of everolimus in the treatment of neurocognitive problems in tuberous sclerosis (TRON): study protocol for a randomised controlled trial. Trials.

[9] Krueger DA, Sadhwani A, Byars AW, de Vries PJ, Franz DN, Whittemore VH (2017). Everolimus for treatment of tuberous sclerosis complex-associated neuropsychiatric disorders. Ann Clin Transl Neurol.

[10] Overwater IE, Rietman AB, Mous SE, Bindels-de Heus K, Rizopoulos D, Ten Hoopen LW, et al. A randomized controlled trial with everolimus for IQ and autism in tuberous sclerosis complex. Neurology. 2019. 10.1212/WNL.0000000000007749 31217257

[11] Talos DM, Sun H, Kosaras B, Joseph A, Folkerth RD, Poduri A (2012). Altered inhibition in tuberous sclerosis and type IIb cortical dysplasia. Ann Neurol.

[12] Ben-Ari Y (2017). NKCC1 Chloride Importer Antagonists Attenuate Many Neurological and Psychiatric Disorders. Trends Neurosci.

[13] Ruffolo G, Iyer A, Cifelli P, Roseti C, Muhlebner A, van Scheppingen J (2016). Functional aspects of early brain development are preserved in tuberous sclerosis complex (TSC) epileptogenic lesions. Neurobiol Dis.

[14] Loscher W, Puskarjov M, Kaila K (2013). Cation-chloride cotransporters NKCC1 and KCC2 as potential targets for novel antiepileptic and antiepileptogenic treatments. Neuropharmacology..

[15] Lozovaya N, Gataullina S, Tsintsadze T, Tsintsadze V, Pallesi-Pocachard E, Minlebaev M (2014). Selective suppression of excessive GluN2C expression rescues early epilepsy in a tuberous sclerosis murine model. Nature Commun.

[16] Kharod SC, Kang SK, Kadam SD (2019). Off-Label use of bumetanide for brain disorders: an overview. Front Neurosci.

[17] Schulte JT, Wierenga CJ, Bruining H (2018). Chloride transporters and GABA polarity in developmental, neurological and psychiatric conditions. Neurosci Biobehav Rev.

[18] Eftekhari S, Mehvari Habibabadi J, Najafi Ziarani M, Hashemi Fesharaki SS, Gharakhani M, Mostafavi H (2013). Bumetanide reduces seizure frequency in patients with temporal lobe epilepsy. Epilepsia.

[19] Kahle KT, Barnett SM, Sassower KC, Staley KJ (2009). Decreased seizure activity in a human neonate treated with bumetanide, an inhibitor of the Na(+)-K(+)-2Cl(-) cotransporter NKCC1. J Child Neurol.

[20] Northrup H, Krueger DA (2013). International Tuberous Sclerosis Complex Consensus G. Tuberous sclerosis complex diagnostic criteria update: recommendations of the 2012 International Tuberous Sclerosis Complex Consensus Conference. Pediatr Neurol.

[21] Lemonnier E, Villeneuve N, Sonie S, Serret S, Rosier A, Roue M (2017). Effects of bumetanide on neurobehavioral function in children and adolescents with autism spectrum disorders. Transl Psychiatry.

[22] Aman MG, Singh NN, Stewart AW, Field CJ (1985). The aberrant behavior checklist: a behavior rating scale for the assessment of treatment effects. Am J Mental Deficiency.

[23] Constantino JN, Davis SA, Todd RD, Schindler MK, Gross MM, Brophy SL (2003). Validation of a brief quantitative measure of autistic traits: comparison of the social responsiveness scale with the autism diagnostic interview-revised. J Autism Dev Disord.

[24] Dunn W (1999). Sensory profile: User's manual: Psychological Corporation San Antonio, TX.

[25] Dunn W (2006). Sensory profile-school companion manual.

[26] Lam KS, Aman MG (2007). The Repetitive behavior scale-revised: independent validation in individuals with autism spectrum disorders. J Autism Dev Disord.

[27] Gioia GA, Isquith PK, Guy SC, Kenworthy L (2015). BRIEF-2: Behavior rating inventory of executive function: professional manual: psychological assessment resources.

[28] Group W (1998). Development of the World Health Organization WHOQOL-BREF quality of life assessment. Psychological medicine..

[29] Herdman M, Gudex C, Lloyd A, Janssen M, Kind P, Parkin D (2011). Development and preliminary testing of the new five-level version of EQ-5D (EQ-5D-5 L). Qual Life Res.

[30] Wille N, Badia X, Bonsel G, Burström K, Cavrini G, Devlin N (2010). Development of the EQ-5D-Y: a child-friendly version of the EQ-5D. Qual Life Res.

[31] Varni JW, Seid M, Kurtin PS (2001). PedsQL 4.0: reliability and validity of the Pediatric Quality of Life Inventory version 4.0 generic core scales in healthy and patient populations. Medical Care.

[32] Vlaskamp C, Oranje B, Madsen GF, Møllegaard Jepsen JR, Durston S, Cantio C (2017). Auditory processing in autism spectrum disorder: Mismatch negativity deficits. Autism Res.

[33] Madsen GF, Bilenberg N, Cantio C, Oranje B (2014). Increased prepulse inhibition and sensitization of the startle reflex in autistic children. Autism Res.

[34] Madsen GF, Bilenberg N, Jepsen JR, Glenthoj B, Cantio C, Oranje B. Normal P50 Gating in Children with Autism, Yet Attenuated P50 Amplitude in the Asperger Subcategory. 2015 (1939-3806 (Electronic)). 10.1002/aur.1452 25599888

[35] de Vries P, Humphrey A, McCartney D, Prather P, Bolton P, Hunt A (2005). Consensus clinical guidelines for the assessment of cognitive and behavioural problems in Tuberous Sclerosis. Eur Child Adolesc Psychiatry.

[36] de Sonneville L (1999). Amsterdam Neuropsychological tasks: a computer-aided assessment program. Cognitive Ergonomics, Clinical Assessment and Computer-assisted Learning: Computers in Psychology..

[37] Cohen J (1988). Statistical power analysis for the behavioral sciences: Routledge.

[38] Lemonnier E, Ben-Ari Y (2010). The diuretic bumetanide decreases autistic behaviour in five infants treated during 3 months with no side effects. Acta Paediatr.

[39] Lemonnier E, Degrez C, Phelep M, Tyzio R, Josse F, Grandgeorge M (2012). A randomised controlled trial of bumetanide in the treatment of autism in children. Transl Psychiatry.

[40] Miyajima M, Ohta K, Hara K, Iino H, Maehara T, Hara M (2011). Abnormal mismatch negativity for pure-tone sounds in temporal lobe epilepsy. Epilepsy Res.

[41] Lopes R, Simões MR, Ferraz L, Leal AJR (2014). The mismatch negativity (MMN) potential as a tool for the functional mapping of temporal lobe epilepsies. Epilepsy Behav.

[42] de Vries PJ, Franz DN, Curatolo P, Nabbout R, Neary M, Herbst F (2018). Measuring Health-related quality of life in tuberous sclerosis complex—psychometric evaluation of three instruments in individuals with refractory epilepsy. Front Pharmacol.

[43] Kharod SC, Kang SK, Kadam SD. Off-Label Use of Bumetanide for Brain Disorders: An Overview. Frontiers in neuroscience. 2019;13:310-. 10.3389/fnins.2019.00310 PMC6491514 31068771

[44] Stunnenberg BC, Raaphorst J, Groenewoud HM, Statland JM, Griggs RC, Woertman W, et al. Effect of mexiletine on muscle stiffness in patients with nondystrophic myotonia evaluated using aggregated N-of-1 trials. (1538-3598 (Electronic)). 10.1001/jama.2018.18020. 10.1001/jama.2018.18020 PMC6583079 30535218

