# Supplementary material for: Effects of bumetanide on neurodevelopmental impairments in patients with tuberous sclerosis complex: an open-label pilot study
Source: Mol Autism. 2020 May 7;11:30. doi: 10.1186/s13229-020-00335-4 (PMC7204231; doi:10.1186/s13229-020-00335-4)
Supplement: Supplementary file 1 — Additional file 1. Supplementary methods. [file 13229_2020_335_MOESM1_ESM.docx]

**ADDITIONAL FILE 1**

**Supplementary methods**

**1. EEG recording**

EEG was recorded from 64 electrodes placed in a cap (10-20 layout) with BioSemi® hardware (Amsterdam, Netherlands). Data were recorded with a sampling rate of 2048Hz. Electrooculography electrodes were placed above and below the right eye and beside both eyes to record eye movement and reference electrodes were located on the left and right mastoid. All auditory stimuli were presented using a computer running Presentation software (soundcard: Creative soundblaster 5.1) and were presented through stereo insert earphones (Eartone ABR).

**2. ERP paradigms**

**PPI**. The PPI paradigm started with 5 minutes of acclimation to a continuous background noise (70dB white noise) after which three blocks of stimuli were superimposed. Blocks 1 and 3 were identical and were used to assess habituation and sensitization of the acoustic startle reflex. These two blocks consisted of eight pulse-alone trials of white noise (115dB, 20ms), instant rise and fall. Block 2 consisted of 50 trials presented in a pseudorandomized order and were used to assess PPI: two intensities prepulse stimuli were used (6 and 15dB above the 70dB background, both with 20ms duration), while also two stimulus onset asynchronies (SOA) of 60 and 120ms were used in the trials. The session consisted of 10 pulse alone and 10 of each prepulse–pulse combination (76dB/60ms, 76dB/120ms, 85dB/60ms, 85dB/120ms) which were presented in a pseudo-randomized order (two of the same trial types were never directly following each other). Intertrial intervals in all blocks were randomized between 10 and 20s. The complete paradigm took approximately 25 min.

**P50 suppression**. The P50 paradigm is comprised of 40 paired clicks (1.5ms in duration and 80dB) presented binaurally and repeated in 3 blocks. Subjects were instructed to count the number of clicks. Interstimulus interval (ISI) was consistently 500ms and click pairs were separated by 10s. The total duration of the P50 suppression task is approximately 21 minutes.

**MMN**. The MMN paradigm consisted of 1800 trials with an intensity of 75dB and an ISI randomly varying between 400-500ms. The task stimuli were subdivided into 1500 trials (83,3%) of standard tones with a frequency of 1000Hz and duration of 50ms; 100 trials (5,5%) of frequency deviant tones (1200Hz, 50ms); duration deviant tones (1000Hz, 100ms) and frequency/duration deviant tones (1200Hz, 100ms). Subjects were asked to ignore the stimuli and watched a muted documentary on a screen in front of them. The total duration is approximately 14 minutes.

**3. EEG processing**

The EEG signals were pre-processed, averaged and analyzed using Brain Electrical Source Analysis (BESA)-software (version 6, MEGIS Software GmbH, Gräfeelfing, Germany). Preprocessing of the data started with resampling from the original 2048Hz to 250Hz for MMN, 500Hz for P50 and 1000Hz for PPI to allow for easier file handling. PPI was epoched at -50 to 250ms, band-pass filtered at 25-250Hz and measured bipolarly from both eye electrodes below the right eye.

P50 and MMN were differently processed since they were analyzed over the scalp electrodes. Electrodes with aberrant signals were manually interpolated or removed when >6 channels were affected and eye-blinks were removed by BESAs internal scripts. Third, the data were epoched (from 100ms prestimulus to 900ms poststimulus for MMN and -100 to 400ms for P50) and corrected for movement (or other paradigm unrelated) artefacts, by removing those epochs from the database that contained amplitude differences between maximum and minimum exceeding 75μV, in the for P50, MMN relevant scoring windows (see below). Only data from the electrodes relevant for this study were analyzed (i.e., where the maximum activity for the ERPs was found): the midline electrodes Cz (for P50), FCz (for freqMMN, durMMN, freqdurMMN) and both eye-electrodes for PPI. P50 and MMN data were band-pass filtered (0.5Hz- 70Hz for P50 suppression data, 0.5Hz- 40Hz for MMN data), and grand average reference was used as a reference.

For PPI, startle magnitude was scored as the highest absolute amplitude within a window between 20 and 120ms following the startle eliciting pulse, whereas PPI was expressed as [(1 − (PP/PA)) × 100%]; with PP = the average startle amplitude to prepulse–pulse trials, and PA = the average startle amplitude to pulse alone trials in block 2. Habituation was defined as the β-coefficient of the linear trend line through the points of trials 4–8 in block 1 and trials 1–8 in block 3.

For P50, the amplitude was defined as the largest trough to peak amplitude within an interval of 40–90ms following the first (S1) stimulus in each paired click. The P50 amplitude following the second (S2) stimulus was identified as the largest trough to peak amplitude within an interval of 10ms of the latency of the maximum P50 amplitude to the C-stimulus. P50 suppression was expressed as the ratio “S2/S1”. P50 waves were manually scored by BO and JS.

For MMN, amplitude and mean amplitude were scored for each of the three deviant types, expressed as the average ERP to the relevant deviant stimuli, subtracted with the average ERP to standard stimuli for each subject separately. MMN amplitudes were then scored as the minimum amplitude in a window between 75 and 200ms for frequency and the combined frequency-duration deviants, 180 and 340sec for standard tones and in a window between 100-270ms for duration deviants.
