# Supplementary material for: Effects of bumetanide on neurodevelopmental impairments in patients with tuberous sclerosis complex: an open-label pilot study
Source: Mol Autism. 2020 May 7;11:30. doi: 10.1186/s13229-020-00335-4 (PMC7204231; doi:10.1186/s13229-020-00335-4)
Supplement: Supplementary file 2 — Additional file 2. Neuropsychological test battery results. [file 13229_2020_335_MOESM2_ESM.docx]

**ADDITIONAL FILE 2**

**Neuropsychological test battery results**

**Table 1.** Raw scores on auditory and visual memory tasks at baseline and after 3 months bumetanide treatment.

|  | 15-wt | | | | | | | | RVDLT | | | | WISC-DS | | | | | |
| --- | --- | --- | --- | --- | --- | --- | --- | --- | --- | --- | --- | --- | --- | --- | --- | --- | --- | --- |
|  | Total recall | | Trial 1-5 | | Trial 1 | | DR | | Trial 1-5 | | Recognition | | Forwards | | Backwards | | Total | |
|  | **D0** | **D91** | **D0** | **D91** | **D0** | **D91** | **D0** | **D91** | **D0** | **D91** | **D0** | **D91** | **D0** | **D91** | **D0** | **D91** | **D0** | **D91** |
|  | 27 | 25 | 40 | 44 | 2 | 4 | 10 | 15 | 30 | 46 | 35 | 35 | 10 | 7 | 7 | 3 | 17 | 10 |
|  | 30 | 30 | 50 | 50 | 6 | 3 | 11 | 9 | 40 | 36 | 44 | 40 | 8 | 8 | 4 | 6 | 12 | 14 |
|  | 30 | 30 | 58 | 49 | 3 | 5 | 8 | 11 | 29 | 36 | 39 | 45 | 9 | 11 | 3 | 7 | 12 | 18 |
|  | 30 | 30 | 41 | 48 | 4 | 4 | 6 | 11 | 38 | 41 | 41 | 40 | 8 | 8 | 3 | 4 | 11 | 12 |
|  | 22 | 28 | 38 | 35 | 2 | 2 | 5 | 3 | 19 | 13 | 34 | 30 | 6 | 5 | 2 | 2 | 8 | 7 |
|  | 30 | 30 | 49 | 63 | 6 | 5 | 12 | 10 | 43 | 37 | 42 | 45 | 10 | 8 | 6 | 6 | 16 | 14 |
|  | 29 | 29 | 43 | 41 | 6 | 3 | 11 | 11 | 39 | 40 | 39 | 43 | 7 | 6 | 4 | 3 | 11 | 9 |
| M | 28 | 29 | 46 | 47 | 4 | 4 | 9 | 10 | 34 | 36 | 39 | 40 | 8 | 8 | 4 | 4 | 12 | 12 |

*Abbreviations: 15-WT: 15 Word Task; RVDLT: Rey Visual Design Learning Test; WISC-DS: Wechsler Intelligence Scale for Children-Digit Span; DR: Delayed Recall; M: Mean; WNV-SSp: Wechsler Non-Verbal Scale of Ability subtest Spatial Span*

|  | **WNV-SSp** | | | | | |
| --- | --- | --- | --- | --- | --- | --- |
|  | Forwards | | Backwards | | Total | |
|  | **D0** | **D91** | **D0** | **D91** | **D0** | **D91** |
|  | 6 | 7 | 2 | 3 | 8 | 10 |
|  | 10 | 6 | 8 | 7 | 18 | 13 |
|  | 8 | 6 | 8 | 6 | 16 | 12 |
|  | 6 | 5 | 4 | 6 | 10 | 11 |
|  | 6 | 4 | 2 | 2 | 8 | 6 |
|  | 5 | 5 | 5 | 8 | 10 | 13 |
|  | 6 | 6 | 6 | 6 | 12 | 12 |
| **M** | 7 | 6 | 5 | 5 | 12 | 11 |

| Table 2. Raw scores on the Amsterdam Neuropsychological Test (ANT) battery at baseline and after 3 months bumetanide treatment. | | | | | | | | | | | | | | | | | | | | | | | | | | | | | | | | | | | | | | | | | | | | | | | | | | | | | | | | |
| --- | --- | --- | --- | --- | --- | --- | --- | --- | --- | --- | --- | --- | --- | --- | --- | --- | --- | --- | --- | --- | --- | --- | --- | --- | --- | --- | --- | --- | --- | --- | --- | --- | --- | --- | --- | --- | --- | --- | --- | --- | --- | --- | --- | --- | --- | --- | --- | --- | --- | --- | --- | --- | --- | --- | --- | --- |
|  | | **Baseline speed** | | | | | | | | | | | | | | | | | | | | | | | | | | | | **Go No-go** | | | | | | | | | | | | | | | | | | | | | | | | | |  |
|  | | RT (ms) | | | | | | | | | | | | Stability (ms) | | | | | | RT (ms) | | | | | | | | | Missed | | | | | | | | FA | | | | | | | | RT Bias (ms) | | | | | Missed Bias | | | FA Bias | | |  |
|  | | **D0** | | | | | | | **D91** | | | | **D0** | | **D91** | | | | | | **D0** | | | **D91** | | | | | | **D0** | | **D91** | | | | **D0** | | | | | | **D91** | | **D0** | | | | **D91** | | | **D0** | **D91** | | **D0** | **D91** |  |
|  | | 225 | | | | | | | 248 | | | | 46 | | 75 | | | | | | 383 | | | 347 | | | | | | 0 | | 0 | | | | 1 | | | | | | 0 | | 382 | | | | 321 | | | 0 | 0 | | 2 | 1 |  |
|  | | 253 | | | | | | | 264 | | | | 45 | | 55 | | | | | | 305 | | | 302 | | | | | | 0 | | 0 | | | | 0 | | | | | | 0 | | 358 | | | | 390 | | | 0 | 0 | | 0 | 2 |  |
|  | | 336 | | | | | | | 315 | | | | 191 | | 163 | | | | | | 474 | | | 484 | | | | | | 0 | | 0 | | | | 1 | | | | | | 0 | | 468 | | | | 494 | | | 0 | 2 | | 1 | 0 |  |
|  | | 324 | | | | | | | 346 | | | | 172 | | 111 | | | | | | 381 | | | 394 | | | | | | 0 | | 0 | | | | 4 | | | | | | 0 | | 376 | | | | 413 | | | 0 | 0 | | 5 | 0 |  |
|  | | 395 | | | | | | | 412 | | | | 196 | | 156 | | | | | | 548 | | | 502 | | | | | | 1 | | 0 | | | | 5 | | | | | | 7 | | 452 | | | | 560 | | | 0 | 1 | | 6 | 7 |  |
|  | | 394 | | | | | | | 312 | | | | 443 | | 91 | | | | | | 412 | | | 353 | | | | | | 0 | | 0 | | | | 0 | | | | | | 0 | | 427 | | | | 384 | | | 0 | 0 | | 0 | 1 |  |
|  | | 372 | | | | | | | 322 | | | | 169 | | 80 | | | | | | 354 | | | 377 | | | | | | 0 | | 0 | | | | 0 | | | | | | 0 | | 354 | | | | 349 | | | 0 | 0 | | 1 | 2 |  |
| M (SD) | | 328 (67) | | | | | | | 317 (54) | | | | 180 (133) | | 104 (41) | | | | | | 408 (80) | | | 394 (73) | | | | | | .14 | | 0 | | | | 1.57 | | | | | | 1 | | 402 (46) | | | | 416 (84) | | | 0 | 0.43 | | 2.14 | 1.86 |  |
|  |  |  |  |  |  |  |  |  |  |  |  |  |  |  |  |  |  |  |  |  |  |  |  |  |  |  |  |  |  |  |  |  |  |  |  |  |  |  |  |  |  |  |  |  |  |  |  |  |  |  |  |  |  |  |  |  |
|  | | | **SSA (ms)** | | | | | | | | | | | | | | | | | | | | | | | | | | | | | | | | | | | | | | | | | | | | | |  |  |  |  |  |  |  |  |
|  | | | | | 1 | | | | | | | | | | | 2 | | | | | | | | | | 3 Compatible | | | | | | | | | | | | 3 Incompatible | | | | | | | | | | |  |  |  |  |  |  |  |  |
|  | | | | **D0** | | | | | | | **D91** | | | | | **D0** | | | | | | **D91** | | | | | | **D0** | | | | | **D91** | | | | | | **D0** | | | | | | | **D91** | | |  |  |  |  |  |  |  |  |
|  | | | | 351 | | | | | | | 414 | | | | | 463 | | | | | | 625 | | | | | | 1241 | | | | | 962 | | | | | | 1160 | | | | | | | 912 | | |  |  |  |  |  |  |  |  |
|  | | | | 358 | | | | | | | 503 | | | | | 620 | | | | | | 674 | | | | | | 774 | | | | | 765 | | | | | | 833 | | | | | | | 986 | | |  |  |  |  |  |  |  |  |
|  | | | | 707 | | | | | | | 483 | | | | | 950 | | | | | | 580 | | | | | | 1048 | | | | | 1016 | | | | | | 1201 | | | | | | | 1288 | | |  |  |  |  |  |  |  |  |
|  | | | | 528 | | | | | | | 501 | | | | | 752 | | | | | | 688 | | | | | | 868 | | | | | 851 | | | | | | 884 | | | | | | | 825 | | |  |  |  |  |  |  |  |  |
|  | | | | - | | | | | | | 704 | | | | | - | | | | | | 1378 | | | | | | - | | | | | 727 | | | | | | - | | | | | | | 1147 | | |  |  |  |  |  |  |  |  |
|  | | | | 383 | | | | | | | 392 | | | | | 665 | | | | | | 595 | | | | | | 1038 | | | | | 818 | | | | | | 1171 | | | | | | | 1026 | | |  |  |  |  |  |  |  |  |
|  | | | | 373 | | | | | | | 357 | | | | | 604 | | | | | | 579 | | | | | | 935 | | | | | 724 | | | | | | 991 | | | | | | | 811 | | |  |  |  |  |  |  |  |  |
| M  (SD) | | | | 450  (142) | | | | | | | 479  (114) | | | | | 676  (164) | | | | | | 731  (288) | | | | | | 984  (163) | | | | | 838  (114) | | | | | | 1040  (159) | | | | | | | 999  (173) | | |  |  |  |  |  |  |  |  |
|  | | | | | | |  |  | |  | | | | | | | | |  | | | | | |  | | | | | |  | | | |  | | | | | | | |  | | | | | |  |  |  |  |  |  |  |  |
|  | | | | | | | **SSV (ms)** | | | | | | | | | | | | | | | | | | | | | | | | | | | | | | | | | | | | | | | | | |  |  |  |  |  |  |  |  |
|  | | | | | | | 1 | | | | | | | | | | 2 | | | | | | | | | | 3 Compatible | | | | | | | | | | | | | | 3 Incompatible | | | | | | | |  |  |  |  |  |  |  |  |
|  | | | | | | **D0** | | | | | | **D91** | | | | | | **D0** | | | | | **D91** | | | | | | | **D0** | | | | **D91** | | | | | | **D0** | | | | | | | **D91** | |  |  |  |  |  |  |  |  |
|  | | | | | | 523 | | | | | | 336 | | | | | | 1175 | | | | | 550 | | | | | | | 1190 | | | | 1474 | | | | | | 951 | | | | | | | 1334 | |  |  |  |  |  |  |  |  |
|  | | | | | | 473 | | | | | | 523 | | | | | | 969 | | | | | 751 | | | | | | | 1363 | | | | 1500 | | | | | | 1520 | | | | | | | 1864 | |  |  |  |  |  |  |  |  |
|  | | | | | | 575 | | | | | | 465 | | | | | | 1125 | | | | | 1085 | | | | | | | 1263 | | | | 1194 | | | | | | 1758 | | | | | | | 1148 | |  |  |  |  |  |  |  |  |
|  | | | | | | 667 | | | | | | 553 | | | | | | 729 | | | | | 890 | | | | | | | 945 | | | | 947 | | | | | | 1073 | | | | | | | 976 | |  |  |  |  |  |  |  |  |
|  | | | | | | 1011 | | | | | | 566 | | | | | | 1218 | | | | | 601 | | | | | | | 1946 | | | | 710 | | | | | | 1200 | | | | | | | 1024 | |  |  |  |  |  |  |  |  |
|  | | | | | | 565 | | | | | | 557 | | | | | | 914 | | | | | 1022 | | | | | | | 1931 | | | | 1613 | | | | | | 2024 | | | | | | | 2045 | |  |  |  |  |  |  |  |  |
|  | | | | | | 502 | | | | | | 544 | | | | | | 2092 | | | | | 1090 | | | | | | | 1523 | | | | 1515 | | | | | | 2000 | | | | | | | 1657 | |  |  |  |  |  |  |  |  |
| M  (SD) | | | | | | 617  (185) | | | | | | 506  (82) | | | | | | 1175  (439) | | | | | 856  (226) | | | | | | | 1452  (376) | | | | 1279  (340) | | | | | | 1504  (441) | | | | | | | 1435  (424) | |  |  |  |  |  |  |  |  |
