# Supplementary material for: Effects of bumetanide on neurodevelopmental impairments in patients with tuberous sclerosis complex: an open-label pilot study
Source: Mol Autism. 2020 May 7;11:30. doi: 10.1186/s13229-020-00335-4 (PMC7204231; doi:10.1186/s13229-020-00335-4)
Supplement: Supplementary file 3 — Additional file 3. Blood safety checks. [file 13229_2020_335_MOESM3_ESM.docx]

**ADDITIONAL FILE 3**

**Blood safety checks**

| **Table 3.** Blood safety checks: potassium | | | | | | | | | | | |
| --- | --- | --- | --- | --- | --- | --- | --- | --- | --- | --- | --- |
| **Patient** | | **Baseline** | **D4** | | **D7** | | **D14** | | **D28** | | **D56** |
| 1 | | 4,0 | 3,8 | | 4,2 | | 3,4 | | 3,8 | | 3,8 |
| 2 | | 3,9 | 4,2 | | 3,7 | | 4,1 | | 3,8 | | 4,1 |
| 3 | | 4,7^a^ | 4,0^a^ | | 4,1^a^ | | 4,0^a^ | | 5,2^a^ | | 4,1^a^ |
| - | | 4,3 | 4,0^b^ | | 4,2^b^ | |  | | 3,8^b^ | |  |
| 4 | | 4,2 | 4,1 | | 4,0 | | 4,1 | | 4,3 | | 3,6 |
| 5 | | 4,4 | 3,5^b^ | | 3,9^b^ | | 3,4^b^ | | 3,4^b^ | | 3,5^b^ |
| 6 | | 3,7 | 3,4 | | 3,6 | | 3,4 | | 3,5 | | 3,6 |
| 7 | | 3,9 | 3,8^b^ | | 3,5^b^ | | 3,2^b^ | | 3,9^b^ | | 3,2^b^ |
| 8 | | 4,4 | 4,7 | | 3,7^b^ | | 4,0 | | 3,8 | | 4,4 |
| 9 | | 3,9 | 4,0^b^ | | 3,9^b^ | | 4,2 | | 3,9^b^ | | 4,2^b^ |
| 10 | | 4,1 | 3,4^b^ | | 4,1^b^ | | 3,8^b^ | | 3,8^b^ | | 4,3^b^ |
| - | | 4,9 | 4,4^b^ | | 4,1^b^ | | 4,1^b^ | | 3,6^b^ | | Drop-out |
| 11 | | 4,0 | 3,9 | | 4,0 | | 4,1 | | 4,1 | | 3,9 |
| 12 | | 4,0^b^ | 4,2^b^ | | 3,5^b^ | | 3,7^b^ | | 3,6^b^ | | 3,3^b^ |
| 13 | | 3,9 | 4,4^a^ | | 4,1 | | 3,8 | | 3,9 | | 4,4^a^ |
| *Note: ^a^Capillary finger-prick blood draw; ^b^blood samples at general practitioner.* | | | | | | | | | | | |
| **Table 4.** Blood safety checks: sodium | | | | | | | | | | | |
| **Patient** | | **Baseline** | **D4** | **D7** | | **D14** | | **D28** | | **D56** | |
| 1 | | 137 | 137 | 139 | | 140 | | 136 | | 138 | |
| 2 | | 138 | 137 | 138 | | 139 | | 138 | | 138 | |
| 3 | | 135^a^ | 136^a^ | 132^a^ | | 138^a^ | | 140^a^ | | 137^a^ | |
| - | | 138 | 139^b^ | 140^b^ | |  | | 142^b^ | |  | |
| 4 | | 138 | 140 | 136 | | 139 | | 138 | | 135 | |
| 5 | | 137 | 141^b^ | 141^b^ | | 142^b^ | | 141^b^ | | 142^b^ | |
| 6 | | 139 | 140 | 139 | | 139 | | 142 | | 143 | |
| 7 | | 136 | 142^b^ | 142^b^ | | 141^b^ | | 144^b^ | | 137^b^ | |
| 8 | | 135 | 137 | 138 | | 134 | | 139 | | 137 | |
| 9 | | 137 | 140^b^ | 143^b^ | | 141^b^ | | 141^b^ | | 143^b^ | |
| 10 | | 139 | 143^b^ | 141^b^ | | 148^b^ | | 143^b^ | | 141^b^ | |
| - | | 140 | 140^b^ | 142^b^ | | 140^b^ | | 142^b^ | | Drop-out | |
| 11 | | 136 | 137 | 137 | | 137 | | 137 | | 136 | |
| 12 | 139^b^ | | 142^b^ | 144^b^ | | 144^b^ | | 142^b^ | | 141^b^ | |
| 13 | 137 | | 137^a^ | 138 | | 139 | | 140 | | 138^a^ | |
| *Note: ^a^Capillary finger-prick blood draw; ^b^blood samples at general practitioner.* | | | | | | | | | | | |
