# Supplementary material for: Effects of bumetanide on neurodevelopmental impairments in patients with tuberous sclerosis complex: an open-label pilot study
Source: Mol Autism. 2020 May 7;11:30. doi: 10.1186/s13229-020-00335-4 (PMC7204231; doi:10.1186/s13229-020-00335-4)

**ADDITIONAL FILE 4**

**Seizure frequency**

Seizure frequency per seizure type as reported by parents 28 days prior to and during treatment phase (D-28 to D91)


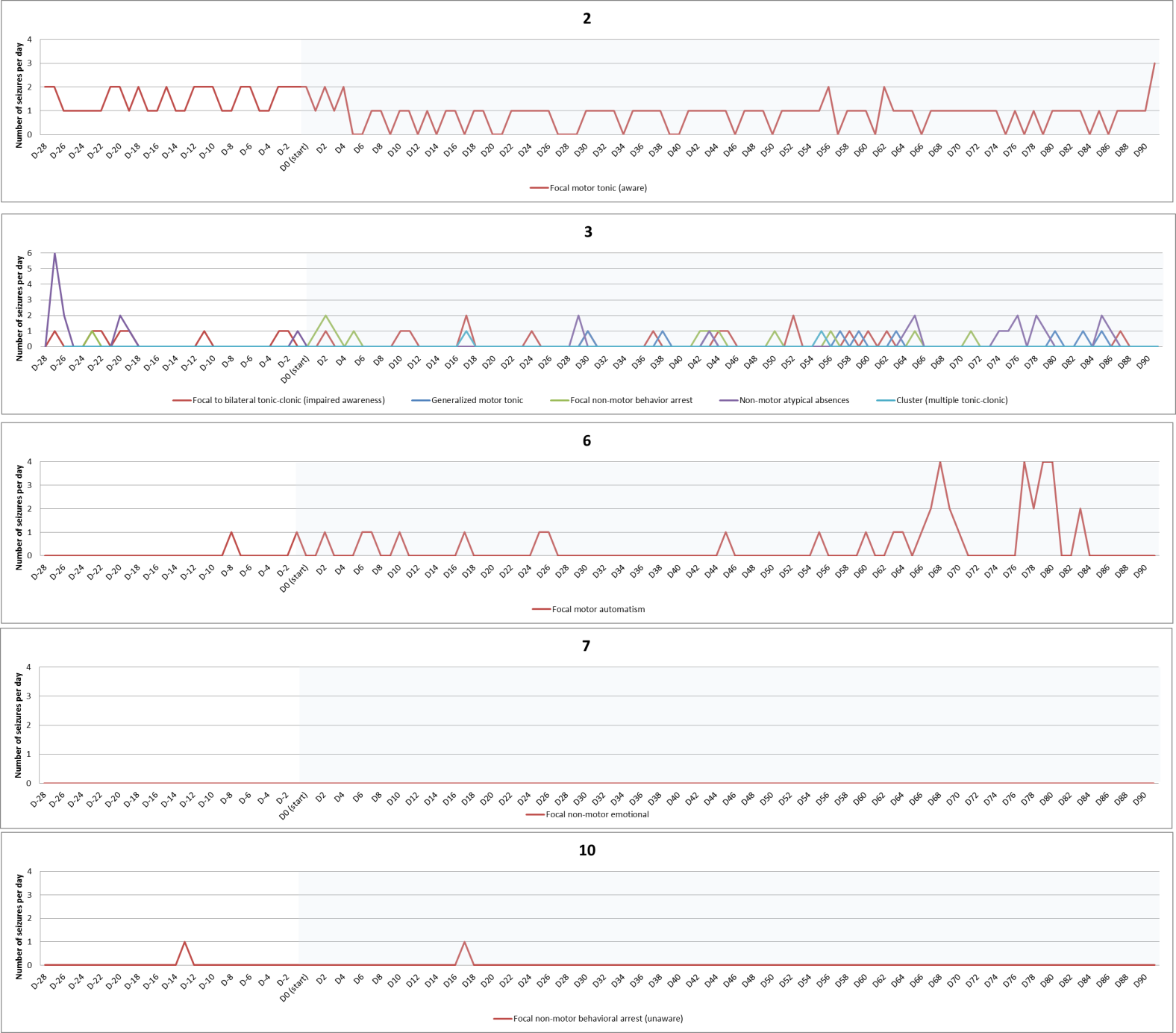

Supplement: Supplementary file 4 — Additional file 4. Seizure frequency. [file 13229_2020_335_MOESM4_ESM.docx]
